# NIMO-CKD-UK: a real-world, observational study of iron isomaltoside in patients with iron deficiency anaemia and chronic kidney disease

**DOI:** 10.1186/s12882-020-02180-2

**Published:** 2020-12-10

**Authors:** Philip A. Kalra, Sunil Bhandari, Michael Spyridon, Rachel Davison, Sarah Lawman, Ashraf Mikhail, David Reaich, Nick Pritchard, Kieran McCafferty, Jason Moore

**Affiliations:** 1 Salford Royal NHS Foundation Trust, Salford, UK; 2 Hull University Teaching Hospitals NHS Trust, Hull, UK; 3 Pharmacosmos UK, Reading, UK; 4 Sunderland Royal Hospital, Sunderland, UK; 5 Royal Sussex County Hospital, Brighton, UK; 6 Morriston Hospital, Swansea, UK; 7 The James Cook University Hospital, Middlesbrough, UK; 8 Addenbrooke’s University Hospital, Cambridge, UK; 9 Royal London University Hospital, London, UK; 10 Royal Devon and Exeter University Hospital, Exeter, UK

**Keywords:** Anaemia, Chronic kidney disease, Ferric derisomaltose, Intravenous iron, Iron deficiency, Iron isomaltoside 1000, Non-dialysis dependent, Observational, Real-world

## Abstract

**Background:**

Intravenous iron is often used to treat iron deficiency anaemia in non-dialysis chronic kidney disease (ND-CKD), but the optimal dosing regimen remains unclear. We evaluated the impact of high- versus low-dose intravenous iron isomaltoside on the probability of retreatment with intravenous iron in iron-deficient ND-CKD patients.

**Methods:**

This real-world, prospective, observational study collected data from 256 ND-CKD patients treated for anaemia in the UK. Following an initial course of iron isomaltoside, patients were followed for ≥12 months. Iron dose and the need for retreatment were determined at the investigators’ discretion. The primary study outcome was the need for retreatment at 52 weeks compared between patients who received >1000 mg of iron during Course 1 and those who received ≤1000 mg. Safety was evaluated through adverse drug reactions.

**Results:**

The probability of retreatment at Week 52 was significantly lower in the >1000 mg iron group (*n* = 58) versus the ≤1000 mg group (*n* = 198); hazard ratio (95% confidence interval [CI]): 0.46 (0.20, 0.91); *p* = 0.012. Mean (95% CI) haemoglobin increased by 6.58 (4.94, 8.21) g/L in the ≤1000 mg group and by 10.59 (7.52, 13.66) g/L in the >1000 mg group (*p* = 0.024). Changes in other blood and iron parameters were not significantly different between the two groups. Administering >1000 mg of iron isomaltoside saved 8.6 appointments per 100 patients compared to ≤1000 mg. No serious adverse drug reactions were reported. Of the patients who received ≤1000 mg of iron in this study, 82.3% were eligible for a dose >1000 mg.

**Conclusions:**

The >1000 mg iron isomaltoside regimen reduced the probability of retreatment, achieved a greater haemoglobin response irrespective of erythropoiesis-stimulating agent treatment, and reduced the total number of appointments required, compared to the ≤1000 mg regimen. Many of the patients who received ≤1000 mg of iron were eligible for >1000 mg, indicating that there was considerable underdosing in this study.

**Trial registration:**

ClinicalTrials.gov NCT02546154, 10 September 2015.

## Background

Iron deficiency anaemia (IDA) is common in chronic kidney disease (CKD) [1], and studies have shown that the prevalence of anaemia increases with declining kidney function [2, 3]. Untreated anaemia can lead to a reduced quality of life [4], disease progression [5, 6], and adverse clinical outcomes [7].

Intravenous (IV) iron can be used to treat anaemia in non-dialysis CKD (ND-CKD) patients, as oral iron is not tolerated by some individuals, and is less efficacious in the more advanced stages of CKD (CKD stages 4 and 5) [7–9]. However, the optimal IV iron regimen in ND-CKD remains unclear. Guidance for anaemia management from the UK National Institute for Health and Care Excellence (NICE) recommends offering high-dose, low-frequency IV iron therapy to ND-CKD patients, stating that a minimum of 500–1000 mg of iron should achieve iron repletion in most patients [7]. On the other hand, evidence from several large randomised controlled trials (RCTs) indicates that the iron deficit in patients with IDA is often >1000 mg [10, 11].

Data on the effectiveness of different IV iron regimens in ND-CKD patients are limited. However, in the dialysis population, the PIVOTAL study – an RCT of 2141 UK haemodialysis patients – showed that higher doses of IV iron were more effective than lower doses on the primary composite endpoint of non-fatal myocardial infarction, non-fatal stroke, hospitalisation for heart failure, or death from any cause [12]. In the non-dialysis population, the REPAIR-IDA study – an RCT in 2584 ND-CKD patients – showed that higher doses of IV iron (1500 mg ferric carboxymaltose) resulted in greater increases in haemoglobin (Hb) and iron parameters compared to lower doses (1000 mg iron sucrose), with similar safety observations reported between the two groups [13]. Taking this one step further, the FIND-CKD study – an RCT in 626 ND-CKD patients – concluded that targeting high ferritin levels by using higher doses of IV iron, delayed and/or reduced the need for further anaemia management [14]. However, few studies have evaluated the real-world impact of higher versus lower IV iron dosing regimens in the management of anaemia in ND-CKD patients.

Iron isomaltoside 1000/ferric derisomaltose (IIM) (produced by Pharmacosmos A/S, Denmark; marketed as Monofer®/Monoferric®) is an IV iron preparation delivered in single, high doses of up to 20 mg/kg body weight [15]. Data from clinical trials have confirmed the good efficacy and safety profile of IIM in patients with CKD [8, 16–19]. In addition, observational studies in CKD conducted in Germany [20], Scandinavia [21], and the UK [22], have demonstrated that IIM is well tolerated and effective in real-world settings.

The purpose of the ‘Non-Interventional Monofer® (NIMO)-CKD-UK’ study was to investigate the impact of high- versus low-dose IIM on the probability of retreatment with IV iron in ND-CKD patients, across different UK hospitals. This pragmatic, real-world study also evaluated the real-world effectiveness of IIM in UK clinical practice.

## Methods

### Study design and population

Participants were recruited from 11 UK hospitals into a prospective, observational study, conducted between January 2016 and December 2018 (ClinicalTrials.gov registry: NCT02546154). The study population included ND-CKD patients (aged ≥18 years) who were diagnosed with IDA according to local hospital guidelines; the study criteria required that the IDA be considered a consequence of CKD. Generally, hospitals in the UK adhere to NICE or Kidney Disease Improving Global Outcomes (KDIGO) clinical guidelines for the management of anaemia in CKD, which recommend using a combination of serum ferritin and transferrin saturation (TSAT) measurements to diagnose iron deficiency [7, 23].

After an initial course of IIM, patients were followed for ≥52 weeks to capture retreatment with IV iron according to local hospital practice. The decision to retreat with IV iron and the patient-specific dosing regimen were based on Hb and/or iron parameters at the time of retreatment, and were at the discretion of each study investigator, in accordance with local clinical practice. The study terminated once the 52-week observational period had been completed and the last blood sample had been collected from the last patient treated with IIM. Patients who reached the end of the 52-week follow-up period having received at least two courses of IV iron completed the study. The remaining patients were followed beyond Week 52 until a second course of IV iron occurred or until study termination. Consequently, the observation period varied between patients and, for some patients, was longer than 52 weeks. Patients were discontinued if they became haemodialysis-dependent, or if they received an alternative IV iron therapy during the study course.

### Data collection and outcome measures

Data for IV iron doses and for blood and iron parameters were collected from patient medical records. Blood samples were taken before IV iron administration and 4–6 weeks after treatment, according to local clinical practice. Patient data were used to estimate iron need using the simplified table and the Ganzoni formula (iron need = patient weight x [target Hb – current Hb] × 0.24 + 500) [24], to allow for a comparison between the estimated iron need and the dose of iron that the patient received (both methods are outlined in the product label for IIM [15]). Quality of life (fatigue symptoms) was assessed before IV iron administration and approximately 4 weeks after treatment, using the 13-item Functional Assessment of Chronic Illness Therapy-Fatigue (FACIT-Fatigue) scale (scored from 0 to 52; higher score indicates better quality of life) [25].

The primary study outcome was the probability of retreatment with IV iron, according to local hospital practice, at Week 52. Secondary outcomes included the probability of retreatment at 6-monthly intervals, the mean change from baseline in blood/iron parameters and in the FACIT-Fatigue questionnaire, and the proportion of patients with an Hb level of ≥110 g/L (the threshold for anaemia investigation and management in CKD patients, according to the NICE guideline) [7]. Exploratory outcomes included a breakdown of the Hb data according to treatment with erythropoiesis-stimulating agents (ESAs) at baseline, and the number of appointments required in each dose group. All adverse drug reactions (ADRs) were reported to the Sponsor’s pharmacovigilance department, and in accordance with the national reporting systems.

### Statistical methods

Data analyses were conducted for all patients who received IIM during the initial treatment course. Analyses were conducted based on comparisons between two dose groups, according to the amount of iron received during the first treatment course (Course 1) – ≤1000 mg or >1000 mg. Selected baseline demographics and clinical characteristics were compared between the groups using a two-sided, two sample t-test assuming unequal variability (weight, kidney function, Hb, ferritin, TSAT, platelets, ESA dose, and FACIT-Fatigue Total score) or a two-sided Fisher’s Exact Test (gender). The primary outcome – probability of retreatment with IV iron at Week 52 – was analysed using a Cox proportional hazards model, with dose group as a factor, and baseline Hb as a covariate. Patients who received only one IV iron course were censored at study end. Data on the primary outcome from patients who discontinued due to receiving an alternative IV iron therapy were included in the primary outcome analysis. The two-sided 95% confidence interval (CI) for the difference in probability of retreatment between the dose groups was calculated and the corresponding *p*-value was derived (based on the pooled standard error estimated from the Cox proportional hazards model). Mean change estimates for blood/iron parameters and FACIT-Fatigue questionnaire before and after treatment were obtained from an analysis of covariance (ANCOVA) model with dose group as a factor, and pre-treatment value as a covariate. Comparisons between groups from the ANCOVA model were two-sided. For the proportion of patients with an Hb level ≥ 110 g/L, odds ratios were calculated, and two-sided *p*-values were obtained from a Fisher’s Exact Test. The significance cut-off for all analyses was *p* < 0.05. No formal statistical analyses were performed on the exploratory outcomes. Missing data were not accounted for in the analyses.

Safety was evaluated through the reporting of ADRs.

## Results

### Patient population

**Fig. 1:**
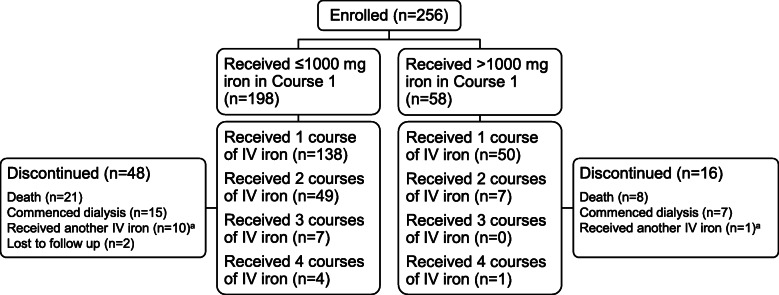
Patient disposition. ^a^Protocol deviation. *IV* intravenous

A total of 256 patients were recruited into the study; 77.3% of patients received ≤1000 mg in Course 1 (Fig. 1).

Patient demographics and baseline clinical characteristics are presented in Table 1.

**Table 1:** Patient demographics and baseline clinical characteristics

	≤1000 mg (***N*** = 198)	> 1000 mg (***N*** = 58)	***p***-value
**Demographics**			
Male, n (%)	96 (48.5)	38 (65.5)	0.025
Weight, kg			
Mean (SD)	77.1 (20.07)	89.7 (19.40)	< 0.001
≥ 50 kg, n (%)	179 (90.4)	58 (100.0)	
≥ 75 kg, n (%)	94 (47.5)	47 (81.0)	
**Clinical characteristics**			
eGFR, mL/min/1.73 m^2^			
Mean (SD)	25.4 (32.37)	25.7 (11.93)	0.910
> 30, n (%)	39 (19.7)	13 (22.4)	
≥ 15 to ≤30, n (%)	122 (61.6)	38 (65.5)	
< 15, n (%)	37 (18.7)	7 (12.1)	
Hb, g/L (n)	(198)	(57)	
Mean (SD)	100.6 (11.65)	102.5 (10.92)	0.263
Ferritin, μg/L (n)	(157)	(45)	
Mean (SD)	161.9 (188.74)	111.9 (103.19)	0.022
Median (Q1, Q3)	106.0 (52.0, 170.0)	68.0 (39.0, 137.0)	
TSAT, % (n)	(97)	(31)	
Mean (SD)	15.8 (6.58)	13.7 (6.03)	0.108
Platelets, × 10^9^/L (n)	(143)	(45)	
Mean (SD)	249.9 (88.12)	222.1 (96.99)	0.091
Concomitant ESA, IU^a^			
Receiving ESA, n (%)	43 (21.7)	15 (25.9)	
Cumulative monthly ESA dose^b^			
Mean (SD)	9505 (12907)	7570 (4963)	0.419
Median	6000	7000	
FACIT-Fatigue Total score (n)	(196)	(58)	
Mean (SD)	25.5 (13.71)	24.1 (13.06)	0.491

*eGFR* estimated glomerular filtration rate, *ESA* erythropoiesis-stimulating agent, *FACIT* Functional Assessment of Chronic Illness Therapy, *Hb* haemoglobin, *IU* international unit, *n* number of patients with data, *Q1* lower quartile of the interquartile range, *Q3* upper quartile of the interquartile range, *SD* standard deviation, *TSAT* transferrin saturation

^a^ESA were administered either intravenously or subcutaneously. Doses expressed in μg were converted to IU (dose in IU/L = dose in μg/L × 200) [12, 26]

^b^Recorded for the 4 weeks prior to study entry

The proportion of male patients was significantly higher in the >1000 mg dose group than ≤1000 mg dose group (*p* = 0.025). Patients in the >1000 mg dose group were significantly heavier than patients in the ≤1000 mg dose group (*p* < 0.001). Baseline kidney function, Hb, TSAT and platelets were similar between the two dose groups. Additional blood and iron parameters were also comparable between the groups (Additional file 1, Table S1; no formal statistical analyses were performed). Ferritin was significantly higher in the ≤1000 mg dose group (*p* = 0.022). The proportion of patients not receiving ESAs was similar between the two groups (≤1000 mg: 78.3%; >1000 mg: 74.1%); of those patients who were receiving ESA the mean cumulative monthly dose of ESA was numerically higher in patients receiving ≤1000 mg IIM.

### Iron dosing and treatment routine

The patient data collected were used to estimate the iron need in patients using the simplified table or the Ganzoni formula [15, 24]. Based on these calculations, the actual dose of IIM administered during Course 1 in the ≤1000 mg group was approximately half of the iron requirement estimated, whereas in the >1000 mg group, the actual dose received was close to the estimated iron need (Table 2). Of the patients who received ≤1000 mg, 82.3% were eligible to receive a dose >1000 mg based on their baseline Hb and weight (Table 2).

**Table 2:** Dose of IIM administered during Course 1 – actual dose versus the estimated iron need

	≤1000 mg (***N*** = 198)		> 1000 mg (***N*** = 58)	
Actual dose received, mg	814.4 (215.53)		1537.9 (185.27)	
Estimated iron need, mg	Simplified	Ganzoni^a^	Simplified	Ganzoni^a^
	1539.1 (341.23)^b^	1410.1 (305.71)^c^	1649.1 (249.37)^d^	1521.4 (318.41)^d^
Difference between actual and estimated dose, mg	-698.3 (387.95)^b^	-581.7 (335.71)^c^	-108.8 (292.94)^d^	19.0 (341.02)^d^
Patients eligible for > 1000 mg of iron, n (%)^e^	163 (82.3)		56 (96.6)	

Data presented are mean (SD)

*n* number of patients with data, *SD* standard deviation

^a^A target Hb of 150 g/L was entered in the Ganzoni formula; ^b^n = 179; ^c^n = 188; ^d^n = 57; ^e^eligibility for > 1000 mg was determined using the simplified dosing table, based on Hb and weight at baseline

Within each dose group, the mean dose of IIM was not affected by whether patients were receiving ESA at baseline or not (Additional file 1, Table S2).

### Probability of retreatment with IV iron

**Fig. 2:**
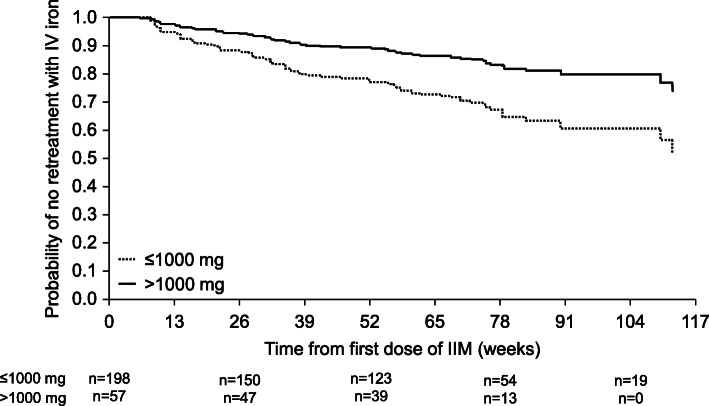
Probability of no retreatment with IV iron, by IIM dose administered during Course 1. *IIM* iron isomaltoside, *IV* intravenous, *n* number of patients included in the analysis

Following a first course of IIM, the probability of no retreatment over time, according to local hospital practice, is presented in Fig. 2.

At Week 52, the probability of retreatment with IV iron was significantly lower in the >1000 mg group than in the ≤1000 mg group – hazard ratio (HR): 0.46 (95% CI: 0.20, 0.91); *p* = 0.012. A statistically significant difference in the probability of retreatment was observed between the two dose groups at all timepoints assessed, up to Week 104 (Additional file 1, Table S3).

### Effectiveness

The change in Hb from baseline to after Course 1 was significantly greater in the > 1000 mg dose group (least squares [LS] mean: 10.59 [95% CI: 7.52, 13.66] g/L) versus the ≤1000 mg group (LS mean: 6.58 [95% CI: 4.94, 8.21] g/L) (*p* = 0.024); no statistically significant difference from baseline was observed after Course 2 (Table 3). Data for additional courses are not presented as very few patients received more than two courses. Within each dose group, the changes in Hb were not affected by ESA treatment at baseline (Additional file 1, Table S4). No significant differences between the two dose groups were observed in the changes before and after treatment for ferritin, TSAT and platelets (Table 3).

**Table 3:** Mean change from baseline in blood and iron parameters before and after IIM treatment

Parameter, by timepoint	≤1000 mg (***N*** = 198)	> 1000 mg (***N*** = 58)	Difference (95% CI)	***p***-value
Hb, g/L				
After Course 1 (≤1000 mg, *n* = 194; > 1000 mg, *n* = 56)	6.58 (4.94, 8.21)	10.59 (7.52, 13.66)	4.02 (0.54, 7.50)	0.024
Before Course 2 (≤1000 mg, n = 56; > 1000 mg, *n* = 10)	-0.26 (-3.61, 3.08)	4.96 (-2.96, 12.88)	5.22 (-3.40, 13.84)	0.231
After Course 2 (≤1000 mg, *n* = 55; > 1000 mg, *n* = 7)	8.39 (4.63, 12.16)	7.79 (-2.85, 18.43)	-0.60 (-11.93, 10.73)	0.916
Ferritin, μg/L				
After Course 1 (≤1000 mg, *n* = 157; > 1000 mg, *n* = 43)	277.98 (238.50, 317.46)	306.09 (227.42, 384.75)	28.10 (-60.32, 116.53)	0.531
Before Course 2 (≤1000 mg, *n* = 45; > 1000 mg, *n* = 7)	91.50 (55.12, 127.88)	120.25 (4.72, 235.77)	28.74 (-93.71, 151.19)	0.638
After Course 2 (≤1000 mg, *n* = 42; > 1000 mg, *n* = 6)	458.23 (317.77, 598.70)	424.37 (-177.2, 1026.0)	-33.86 (-652.3, 584.61)	0.912
TSAT, %				
After Course 1 (≤1000 mg, *n* = 98; > 1000 mg, *n* = 31)	8.13 (5.86, 10.40)	9.90 (5.36, 14.45)	1.77 (-3.33, 6.87)	0.492
Before Course 2 (≤1000 mg, *n* = 35; > 1000 mg, n = 7)	0.89 (-0.95, 2.74)	2.36 (-2.21, 6.94)	1.47 (-3.56, 6.49)	0.555
After Course 2 (≤1000 mg, *n* = 26; > 1000 mg, *n* = 5)	14.89 (8.42, 21.35)	13.17 (-1.60, 27.94)	-1.72 (-18.18, 14.75)	0.830
Platelets, × 10^9^/L				
After Course 1 (≤1000 mg, *n* = 127; > 1000 mg, *n* = 45)	-17.21 (-25.36, -9.06)	-25.75 (-39.47, -12.03)	-8.54 (-24.57, 7.49)	0.294
Before Course 2 (≤1000 mg, *n* = 47; > 1000 mg, *n* = 10)	-0.91 (-17.99, 16.17)	20.25 (-18.25, 58.75)	21.16 (-21.07, 63.38)	0.318
After Course 2 (≤1000 mg, *n* = 49; > 1000 mg, n = 4)	-14.74 (-33.14, 3.67)	-24.17 (-109.5, 61.17)	-9.44 (-96.73, 77.86)	0.828

Data presented are LS mean (95% CI); a negative value indicates a decrease from baseline in the relevant parameter

*CI* confidence interval, *Hb* haemoglobin, *LS* least squares, *n* number of patients with data, *TSAT* transferrin saturation

**Fig. 3:**
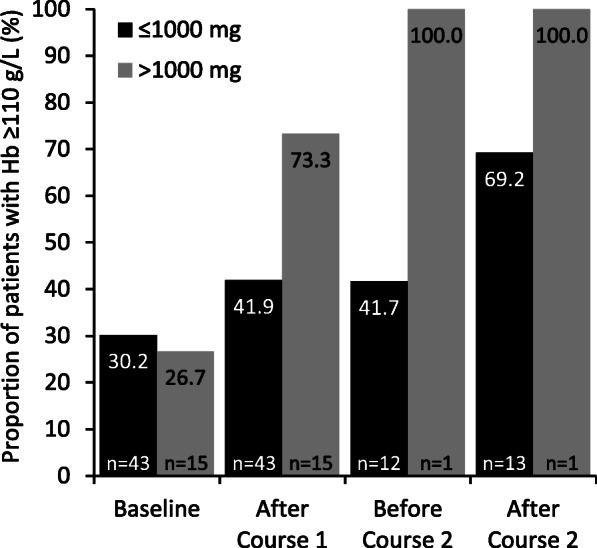
Proportion of patients with Hb ≥110 g/L before and after IIM – patients receiving ESA at baseline. Statistical analyses comparing higher and lower dose groups were performed for baseline and Course 1 only. *ESA* erythropoiesis-stimulating agent, *Hb* haemoglobin, *n* number of patients with data

**Fig. 4:**
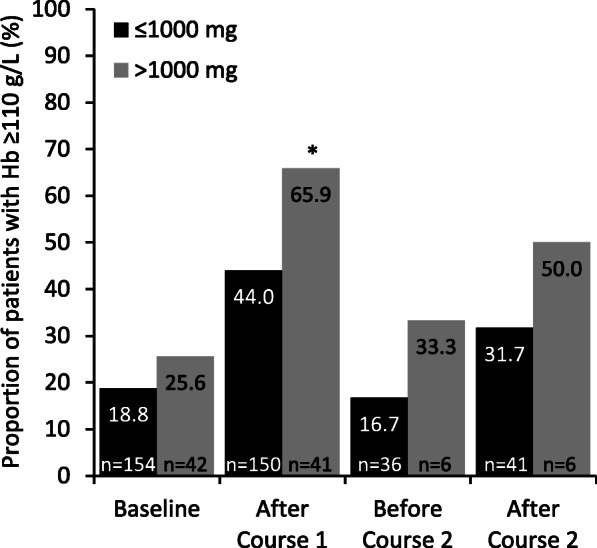
Proportion of patients with Hb ≥110 g/L before and after IIM – patients not receiving ESA at baseline. *p < 0.05 versus ≤1000 mg; statistical analyses comparing higher and lower dose groups were performed for baseline and Course 1 only. *ESA* erythropoiesis-stimulating agent, *Hb* haemoglobin, *n* number of patients with data

After Course 1, the proportion of patients with an Hb level ≥ 110 g/L was greater in the >1000 mg versus the ≤1000 mg group, irrespective of ESA treatment at baseline; the difference was significant in patients not receiving ESA at baseline (*p* = 0.01) (Figs. 3 and 4). A similar trend was observed after Course 2 (Figs. 3 and 4).

Overall, the FACIT-Fatigue score showed a significant improvement from baseline after Course 1, in both dose groups (*p* < 0.0001). The between-group difference in the LS mean change from baseline was not significant (Additional file 1; Table S5).

### Use of healthcare resources

Administering >1000 mg of IIM during Course 1 saved 8.6 appointments per 100 patients compared with administering ≤1000 mg, over the duration of the study (Table 4).

**Table 4:** Number of appointments required, according to the dose of IIM administered in Course 1

	≤1000 mg (***N*** = 198)	> 1000 mg (***N*** = 58)
Total number of appointments	273	75
Appointments per 100 patients	137.9	129.3
Difference between dose groups per 100 patients		−8.6

During Course 1, all patients in the ≤1000 mg group received their prescribed dose in one administration. In the >1000 mg group, the prescribed dose was given in one administration for 86.2% of patients and in two administrations for 13.8% of patients.

### Safety

One non-serious ADR was reported in a total of 336 administrations (0.3%) – pruritus and rash were observed in one patient in the lower dose group. No serious ADRs were reported.

A total of 29 (11.3%) patients died during the study – 21 (10.6%) deaths were reported in the ≤1000 mg group and 8 (13.8%) in the >1000 mg group (Fig. 1). None of the deaths were considered to be related to IIM by the investigators. Overall, there was no difference between the dose groups in the time from the last IV iron infusion to death (≤1000 mg: 19 weeks; >1000 mg: 21 weeks).

## Discussion

The real-world, observational NIMO-CKD-UK study showed that a higher dose IV iron regimen (>1000 mg) decreased the probability of retreatment compared to a lower dose regimen (≤1000 mg). This observation supports the findings of the PIVOTAL, FIND-CKD and REPAIR-IDA studies that higher doses of IV iron can be beneficial [12–14].

NIMO-CKD-UK is a unique study comparing the impact of IV iron doses on the probability of retreatment in non-dialysis patients. When calculated using the simplified table or the Ganzoni formula, most patients requiring IV iron would receive an initial dose >1000 mg. Such doses have been shown to reduce the probability of retreatment for anaemia in indications other than CKD. A Scandinavian observational study showed that gastroenterology patients receiving >1000 mg IIM during an initial course had a 65% lower probability of retreatment at Week 52 versus those receiving 1000 mg [27]. Reducing the need for retreatment with IV iron can have a positive impact on the use of healthcare resources and for patients. Indeed, the findings of the NIMO-CKD-UK study suggest fewer hospital appointments with the use of higher versus lower doses of IIM, which could translate into economic savings and alleviate pressures on healthcare systems that often operate near capacity. Current evidence from health economic analyses suggests that IIM can provide cost-savings to the healthcare system through lower resource usage (lower number of infusions per patient and a lower proportion of patients requiring multiple infusions) compared with other treatments [28–31]. Fewer appointments reduce the burden on patients, who may need to travel long distances to attend appointments. In addition, fewer hospital visits could have clear benefits during the COVID-19 pandemic, where social distancing is necessary and reducing pressure on hospital resources is crucial [32, 33].

Patients receiving >1000 mg IIM during Course 1 also showed a greater Hb response compared with the lower dose group. There was a higher proportion of patients with an Hb level of ≥110 g/L in the > 1000 mg dose group, irrespective of ESA treatment – this is an important observation as the UK NICE guidance recommends the investigation and management of anaemia in patients with an Hb level of <110 g/L [7]. Furthermore, these data support the existing evidence that 1000 mg of IV iron may not be sufficient to achieve iron repletion in CKD patients [11]. Indeed, across all patients, the mean estimated iron need was >1400 mg and, in the ≤1000 mg group, 82.3% of patients were ‘underdosed’. These findings also highlight that the validated methods for estimating iron need are not used in all hospitals, but the clinical impact of this and the reasons why remain unclear. It is possible that underdosing occurred at some hospitals due to the perception that high doses of iron could be harmful; in addition, there is a tendency for a pragmatic attitude to dosing among clinicians, which aims to avoid iron wastage. The NIMO-CKD-UK study also reflects the fact that body weight influences the IV iron dose to some extent, as Hb levels and the proportion of ESA users were similar at baseline between the two dose groups. The results of this study provide support for the use of higher doses; the results show that high doses of IV iron are effective and can be administered without adverse effects.

The use of ESA requires consideration when interpreting the Hb response following IV iron therapy. In this study, a greater Hb response was observed in the higher versus the lower IV iron dose group, irrespective of baseline ESA use. However, at baseline, the monthly cumulative ESA dose was substantially higher in the lower IV iron dose group than in the higher dose group (9505 IU versus 7570 IU); this observation indicates that higher ESA doses were, potentially, used to compensate for the underdosing of iron, or vice versa.

Clinical guidelines recommend maintaining the Hb level in the range of 100–120 g/L in CKD patients receiving ESA treatment [7]. Targeting higher Hb levels with ESA therapy is associated with an increased risk of stroke, hypertension, and vascular access thrombosis, as well as a possible increased risk for death, serious cardiovascular events, and end-stage renal disease [34]. The NIMO-CKD-UK study showed that the mean Hb levels in the >1000 mg IV iron group following Course 1 did not increase beyond 120 g/L, even in patients receiving ESA at baseline, offering reassurance that higher initial doses of IV iron do not elevate Hb to levels that are associated with safety risks (an Hb level > 120 g/L is acceptable for patients not receiving ESA [35]). Indeed, the results of the PIVOTAL study support this theory [12]. More specifically, it has been postulated that ESA-induced iron deficiency leads to an increase in platelet count that can cause thrombocytosis – a risk factor for thrombovascular events [36]. Evidence suggests that IV iron can reduce the platelet count [37], thereby lowering the risk of thrombocytosis [38]. The NIMO-CKD-UK study supports this theory by showing a reduction in platelet count following IV iron treatment, in both dose groups (Table 3). Platelet counts were higher in the lower dose group, possibly a reflection that these patients did not receive their full iron need and retained a certain degree of iron deficiency (known to increase platelet count [36]). The cumulative dose of ESA at baseline was also higher in the lower IV iron dose group.

The observed improvements in FACIT-Fatigue score confirm the established beneficial effect of IV iron on quality of life [39]. A lack of a statistically significant difference between the higher and lower dose groups cannot be explained easily. It may be that the scale is not sensitive enough to capture a difference between the groups, or the improvements in Hb between the two groups may not have been large enough to translate into a differential benefit on quality of life. In addition, it cannot be ruled out that a lack of a detectable difference between the two groups was due to the small patient numbers.

The safety data are consistent with the established profile for IIM showing a low risk of ADRs in patients with CKD from clinical trials and other observational studies [8, 17, 18, 20–22]. For example, the FERWON-NEPHRO study – an RCT of over 1000 IIM-treated patients – reported that IIM was well tolerated with a low ADR rate [19]. In addition, the FERWON-NEPHRO study showed a significantly lower rate of cardiovascular events with IIM versus the comparator, iron sucrose [19]. The proportion of deaths that occurred during the NIMO-CKD-UK study is similar to that reported in a previous large observational study [20], and should be considered within the context of a real-world patient population with chronic illness and multiple morbidities. The time interval between the last IV iron administration and death supports the investigators’ judgement that these events are unrelated to IV iron treatment.

Considering the study limitations, the pragmatic design dictated that no instructions were provided to the investigators on dosing, time to the assessment of treatment response, or how to assess the need for retreatment and, therefore, no information on these practices was collected. Indeed, the baseline data indicate that the tests used to identify iron deficiency (ferritin and/or TSAT) may have varied between centres. However, the aim was to observe treatment routines in clinical practice to provide guidance for optimised strategies for IV iron treatment. In addition, the small number of patients who received a second course of IV iron treatment hinders interpretation of the data collected after repeated dosing, and prevents any conclusions in relation to a dose effect. Although the study did not collect data for ESA therapy post-baseline to determine whether IV iron therapy reduced the need for ESAs, there is no reason to expect that substantial changes in the ESA regimen would have been necessary. It is, therefore, assumed that higher ESA doses were used in the ≤1000 mg group throughout the study course. Finally, this study did not evaluate the longer-term implications of the different doses of IV iron; future studies are needed to examine any such impact by analysing, for example, number of hospitalisations.

## Conclusions

The NIMO-CKD-UK study shows that current clinical practice with IIM is effective in ND-CKD patients. In a real-world setting in the UK, a higher initial dose (>1000 mg) of IIM reduced the probability of retreatment, achieved a greater Hb response irrespective of ESA treatment, and reduced the total number of appointments required compared with a lower dose (≤1000 mg). Use of a higher initial dose treatment approach could be considered to reduce hospital visits during the COVID-19 pandemic.

## Supplementary Information

**Additional file 1:** **Table S1.** Additional blood and iron parameters at baseline. **Table S2.** Dose of IIM administered during Course 1, according to ESA treatment. **Table S3.** Probability of retreatment with IV iron, according to the IIM dose administered during Course 1. **Table S4.** Mean change in Hb from baseline, according to ESA treatment. **Table S5.** Mean change in FACIT-Fatigue Total score from baseline to after Course 1.

## Data Availability

The datasets used and/or analysed during the current study are available from the corresponding author on reasonable request.

## Abbreviations

ADR: adverse drug reaction
ANCOVA: analysis of covariance
CI: confidence interval
CKD: chronic kidney disease
eGFR: estimated glomerular filtration rate
ESA: erythropoiesis-stimulating agent
FACIT: Functional Assessment of Chronic Illness Therapy
Hb: haemoglobin
IDA: iron deficiency anaemia
IIM: iron isomaltoside
IU: international unit
IV: intravenous
KDIGO: Kidney Disease Improving Global Outcomes
LS: least squares
n: number of patients with data
ND-CKD: non-dialysis chronic kidney disease
NICE: National Institute for Health and Care Excellence
NIMO: Non-Interventional Monofer
RCT: randomised controlled trial
SD: standard deviation
TSAT: transferrin saturation
