# Supplementary material for: NIMO-CKD-UK: a real-world, observational study of iron isomaltoside in patients with iron deficiency anaemia and chronic kidney disease
Source: BMC Nephrol. 2020 Dec 10;21:539. doi: 10.1186/s12882-020-02180-2 (PMC7726614; doi:10.1186/s12882-020-02180-2)
Supplement: Supplementary file 1 — Additional file 1: Table S1. Additional blood and iron parameters at baseline. Table S2. Dose of IIM administered during Course 1, according to ESA treatment. Table S3. Probability of retreatment with IV iron, according to the IIM dose administered during Course 1. Table S4. Mean change in Hb from baseline, according to ESA treatment. Table S5. Mean change in FACIT-Fatigue Total score from baseline to after Course 1. [file 12882_2020_2180_MOESM1_ESM.docx]

## Title

NIMO-CKD-UK: a real-world, observational study of iron isomaltoside in patients with iron deficiency anaemia and chronic kidney disease

**Authors and affiliations**

Philip A Kalra;^1^ Sunil Bhandari;^2^ Michael Spyridon;^3^ Rachel Davison;^4^ Sarah Lawman;^5^ Ashraf Mikhail;^6^ David Reaich;^7^ Nick Pritchard;^8^ Kieran McCafferty;^9^ Jason Moore^10^

^1^Salford Royal Hospital, Salford, UK; ^2^Hull University Teaching Hospitals NHS Trust, Hull, UK; ^3^Pharmacosmos UK, Reading, UK; ^4^Sunderland Royal Hospital, Sunderland, UK; ^5^Royal Sussex County Hospital, Brighton, UK; ^6^Morriston Hospital, Swansea, UK; ^7^The James Cook University Hospital, Middlesbrough, UK; ^8^Addenbrooke’s University Hospital, Cambridge, UK; ^9^Royal London University Hospital, London, UK; ^10^Royal Devon and Exeter University Hospital, Exeter, UK

## Additional file 1

**Table S1: Additional blood and iron parameters at baseline**

|  | **≤1,000 mg (N=198)** | **>1,000 mg  (N=58)** |
| --- | --- | --- |
| Iron, µg/dL (n) | (94) | (31) |
| Mean (SD) | 8.6 (3.73) | 8.2 (3.03) |
| CRP, mg/L (n) | (61) | (12) |
| Mean (SD) | 13.2 (16.88) | 13.8 (8.90) |
| MCV, fL (n) | (195) | (58) |
| Mean (SD) | 89.12 (7.775) | 88.48 (5.844) |
| MCH, pg (n) | (189) | (58) |
| Mean (SD) | 28.54 (2.929) | 28.57 (2.390) |
| MCHC, g/L (n) | (122) | (45) |
| Mean (SD) | 317.9 (14.14) | 321.8 (11.33) |

CRP=C-reactive protein; MCH=mean corpuscular haemoglobin; MCHC=mean corpuscular haemoglobin concentration; MCV=mean corpuscular volume; n=number of patients with data; SD=standard deviation

**Table S2: Dose of IIM administered during Course 1, according to ESA treatment**

|  | **≤1,000 mg  (N=198)** | **>1,000 mg  (N=58)** |
| --- | --- | --- |
| Patients receiving ESA (n) | (43) | (15) |
| IIM dose mg (SD) | 702.3 (224.13) | 1,580.0 (207.71) |
| Patients not receiving ESA (n) | (155) | (43) |
| IIM dose mg (SD) | 847.7 (201.82) | 1,523.3 (177.06) |

Data presented are mean (SD)

ESA=erythropoiesis-stimulating agent; IIM=iron isomaltoside; n=number of patients with data; SD=standard deviation

**Table S3: Probability of retreatment with IV iron, according to the IIM dose administered during Course 1**

|  | **≤1,000 mg  (n=198)** | **>1,000 mg  (n=57)** | **p-value** |
| --- | --- | --- | --- |
| Week 26 | 0.125 (n=150) | 0.060 (n=47) | 0.015 |
| Week 52 | 0.232 (n=123) | 0.114 (n=39) | 0.012 |
| Week 78 | 0.342 (n=54) | 0.176 (n=13) | 0.013 |
| Week 104 | 0.407 (n=19) | 0.215 (n=0) | 0.014 |

IIM=iron isomaltoside; IV=intravenous; n=number of patients in the analysis

**Table S4: Mean change in Hb from baseline, according to ESA treatment**

|  | **≤1,000 mg  (N=198)** | **>1,000 mg  (N=58)** | **LS mean difference  (95% CI)** | **p-value** |
| --- | --- | --- | --- | --- |
| **After Course 1** | | | | |
| Patients receiving ESA (n) | (43) | (15) | – | – |
| Hb, g/L, mean (SD) | 107.9 (15.46) | 113.0 (14.58) | – | – |
| Change from baseline, mean (SD) | 4.9 (13.91) | 11.7 (13.86) | – | – |
| LS mean change from baseline (95% CI) | 4.61 (0.24, 8.97) | 11.19 (4.26, 18.12) | 6.59 (-1.60, 14.78) | 0.112 |
| Patients not receiving ESA (n) | (150) | (41) | – | – |
| Hb, g/L, mean (SD) | 107.1 (12.97) | 112.2 (13.16) | – | – |
| Change from baseline, mean (SD) | 7.2 (11.94) | 9.5 (11.31) | – | – |
| LS mean change from baseline (95% CI) | 6.99 (5.19, 8.79) | 10.36 (6.89, 13.82) | 3.37 (-0.54, 7.28) | 0.091 |
| **After Course 2** | | | | |
| Patients receiving ESA (n) | (13) | (1) | – | – |
| Hb, g/L, mean (SD) | 111.2 (8.71) | 130.0 (N/A) | – | – |
| Change from baseline, mean (SD) | 9.4 (15.48) | 11.0 (N/A) | – | – |
| Patients not receiving ESA (n) | (41) | (6) | – | – |
| Hb, g/L, mean (SD) | 107.8 (14.04) | 105.2 (18.52) | – | – |
| Change from baseline, mean (SD) | 9.2 (19.46) | 2.0 (11.98) | – | – |

CI=confidence interval; ESA=erythropoiesis-stimulating agent; Hb=haemoglobin; LS=least squares; N/A=not applicable; SD=standard deviation

**Table S5: Mean change in FACIT-Fatigue Total score from baseline to after Course 1**

|  | **All patients (N=256)** | **≤1,000 mg  (N=198)** | **>1,000 mg  (N=58)** | **LS mean difference  (95% CI)** |
| --- | --- | --- | --- | --- |
| After Course 1 (n) | (213) | (160) | (53) | – |
| Mean (SD) | 32.4 (13.85) | 32.7 (14.43) | 31.3 (11.98) | – |
| Change from baseline, mean (SD) | 7.1 (11.30) | 7.0 (11.72) | 7.6 (10.02) | – |
| p-value | <0.0001 | <0.0001 | <0.0001 | – |
| LS mean change from baseline (95% CI) | – | 7.23 (5.58, 8.87) | 7.10 (4.28, 9.92) | -0.12 (-3.39, 3.15) |
| p-value | – | – | – | 0.941 |

CI=confidence interval; FACIT=Functional Assessment of Chronic Illness Therapy; LS=least squares; n=number of patients with data; SD=standard deviation
